# Epidemiology and health surveillance: synergies and challenges in a changing world

**DOI:** 10.1590/1980-549720250006.supl.1

**Published:** 2026-01-26

**Authors:** Guilherme Loureiro Werneck

**Affiliations:** I Universidade do Estado do Rio de Janeiro Institute of Social Medicine Rio de Janeiro RJ Brazil Universidade do Estado do Rio de Janeiro, Institute of Social Medicine – Rio de Janeiro (RJ), Brazil.; II Universidade Federal do Rio de Janeiro Institute of Studies on Collective Health Rio de Janeiro RJ Brazil Universidade Federal do Rio de Janeiro, Institute of Studies on Collective Health – Rio de Janeiro (RJ), Brazil.; III Ministério da Saúde Department of Health and Environmental Surveillance Brasília DF Brazil Ministério da Saúde, Department of Health and Environmental Surveillance – Brasília (DF), Brazil.

**Keywords:** Public health surveillance, Epidemiology, Uses of epidemiology, Professional training, Health services research

## Abstract

In this essay, we discuss the challenges related to the interaction between epidemiology and health policies, programs, and services, with a particular focus on health surveillance. Although the scope of health surveillance encompasses knowledge beyond epidemiology, the use of epidemiological knowledge is paramount for enhancing the quality of its actions. We present the trajectory of the institutionalization process of health surveillance in Brazil, which culminated in the establishment of the National Health Surveillance Policy (PNVS) in 2018. The PNVS highlights a series of needs that can and should be addressed by epidemiology. These needs offer opportunities for greater interaction between epidemiology and health policies, programs, and services, but they require an update in epidemiology training models, both at the undergraduate and graduate levels, a greater appreciation for knowledge translation activities, and an improved alignment of the academic environment with advances in health surveillance policies in Brazil. Based on the analysis of Brazilian articles on health surveillance listed in the PubMed database, as well as data from research lines, projects, and theses and dissertations from graduate programs, we evaluate how the topic of health surveillance is integrated into research and graduate education in Collective Health in Brazil. We conclude this essay by discussing the contribution of the Fifth Strategic Plan for the Development of Epidemiology in Brazil (2025–2029) for strengthening health surveillance.

## INTRODUCTION

The theoretical framework of epidemiology considers the population distribution of the health-disease process and its determinants and limitations as a particular domain of interest^1,2^. In this context, epidemiology works with theories about the specific mechanisms that cause these events and the reasons that lead to the spatiotemporal heterogeneity of their distribution in populations^2^, a subtle difference that is expressed in multiple practices and perspectives of epidemiology — which, in turn, reflect worldviews prevailing in different times and places^3^.

Acknowledging such diversity implies demarcating the conception of epidemiology here considered: it starts from the principle that epidemiology is a scientific venture that seeks the generation of innovative knowledge and that is committed to its translation and application to subsidize effective actions and policies that lead to the reduction of social inequalities in health and promote a fairer society^2^. An epidemiology that recognizes its roots in the struggles for democratization of the country and for the Health Reform, that is not conformed to only being an epidemiology-science, but also needs to be an epidemiology-action; that is, an epidemiology of Collective Health^4^.

It is precisely in this interface between science and practice that lies one of the greatest challenges for the development of an epidemiology implicated in social transformation. The first obstacle is the very nature and dynamics of public health decision-making. Although the different management levels of the Brazilian Unified Health System (SUS) can recognize that such decisions should be based on the best available scientific evidence, it is noteworthy that they go beyond the scope of science, requiring social, economic, legal, and ethical considerations, as well as values and principles such as equity, transparency, and social participation^5^. Nevertheless, epidemiology has been increasingly urged to contribute to this decision-making process, as observed during the Covid-19 pandemic^6^. This recent experience of having achieved a high degree of protagonism and prestige evidenced the technical and scientific maturity of epidemiology as well as its capillarization in the practices of health institutions. However, it also demonstrated a series of challenges regarding its interaction with health policies, programs, and services^7,8^.

Considering the identities between its definitions and objectives, health surveillance is an unequivocal field of practices for epidemiology. The definition of epidemiology encompasses both the study on the health-disease process and its determinants in populations and the application of this knowledge to control health issues^9^. In turn, health surveillance emphasizes the process of transforming data into information to subsidize the planning and implementation of actions for health protection and promotion and prevention and control of risks, harms, and diseases in populations^10^. Although the scope of health surveillance comprises knowledge beyond epidemiology, epidemiological knowledge is paramount for the qualification of its actions^11^.

### Data availability statement

The entire dataset that supports the results of this study is available upon request to the author.

### Institutionalization of health surveillance in Brazil

At the beginning of the 20th century, even at the international level, surveillance practices did not present significant institutional organization. The term "epidemiological surveillance" began to be adopted in the control of communicable diseases around mid-20th century, initially emphasizing the surveillance of people based on isolation or quarantine measures, but soon expanding its scope to health events in populations. The structuring and institutionalization of services specifically oriented toward the performance of epidemiological surveillance actions gained strength in the international scenario with the creation of the epidemiological surveillance unit in the Communicable Diseases Department of the World Health Organization (1965) and with the technical discussions at the 21st World Health Assembly (1968), which reinforce the understanding of surveillance as an essential function of public health practices^12,13^. In parallel, the International Health Regulations (IHR), created in 1951, and its subsequent amendments, established new processes of monitoring, surveillance, and response to public health emergencies of international importance^14^.

The constitution and institutionalization of health surveillance in Brazil have as a possible milestone the creation of the National Department of Rural Endemic Diseases (*Departamento Nacional de Endemias Rurais* – DNERu), in 1956, which allowed to integrate campaigns against the so-called major endemic diseases, which encompassed epidemiology- and surveillance-related actions^15^. However, only with the creation of the National Epidemiological Surveillance System (*Sistema Nacional de Vigilância Epidemiológica* – SNVE), in 1975, a normative consolidation on the organization of epidemiological surveillance actions in the country was established. Based on vertical and centralized actions in the Federal and State health management spheres, the SNVE reorganized itself as of the 1990s, with the creation and implementation of the SUS and the establishment of the National Center of Epidemiology (*Centro Nacional de Epidemiologia* – CENEPI).

This process advanced in subsequent years, based on regulatory frameworks such as:

Ordinance 1.399/1999 (GM/MS), which establishes the attributions of each government sphere in the area of epidemiology and disease control and defines the financing system^16^;

The creation, in 2003, of the Department of Health Surveillance (*Secretaria de Vigilância em Saúde* – SVS/MS) in order to strengthen and expand the Health Surveillance actions in the Brazilian Ministry of Health;

The Pact for Health (2006), in which the SNVE became known as the National Health Surveillance System (*Sistema Nacional de Vigilância em Saúde* – SNVS);

Ordinance 3.252/2009 (GM/MS), which established the guidelines for the performance and financing of Health Surveillance actions in Brazil, expanding its scope to cover epidemiological, sanitary, occupational health, and environmental health surveillance as well as health promotion and health situation surveillance^17^.

The movement for structuring a Brazilian State policy for health surveillance has been strengthened with the publication of Ordinance 1.378/2013 (GM/MS), which provides for the creation of the Tripartite Work Group for discussion and elaboration of a National Health Surveillance Policy (*Política Nacional de Vigilância em Saúde* – PNVS) — this group, in turn, was put into force the following month^18,19^. Finally, the PNVS was established in 2018 by Resolution 588/2018 of the National Health Council, considering the resolutions of the 1st National Health Surveillance Conference, held in Brasília, Federal District, in the beginning of 2018^10,20^.

### Epidemiology and PNVS

The PNVS is a universal and transversal State public policy that guides the healthcare model in the territories. It establishes the principles, guidelines, and strategies to be observed by the three spheres of SUS management for the development of health surveillance. According to the PNVS, the organization of health surveillance actions comprises the articulation of knowledge, processes, and practices of epidemiological, sanitary, environmental health, and occupational health surveillance. At the same time, the PNVS aims at integrality in health care and presupposes the insertion of health surveillance in the healthcare network. The PNVS encompasses the entire population, but prioritizes territories and populations in situations of greater risk and vulnerability, in such a way to overcome social and health inequalities and seeking equity in care^10^.

This policy results from the struggles for the constitution and defense of the SUS, and the success of its implementation represents the consolidation of a comprehensive vision of health surveillance as a fundamental foundation for the promotion and protection of the health of the population and a structuring axis of Primary Health Care^21-23^.

Although it was established in 2018, there is still no effective implementation of PNVS in the territories. These seven years since the PNVS implementation were particularly challenging. The cycle of fiscal austerity associated with the greater participation of parliamentary amendments in the health budget implied worsening of the already chronic underfunding of the SUS and limitation in the implementation of social policies^24^. Within this context, the impact of the Covid-19 pandemic was dramatic: it had deepened the already substantial social inequalities of the country and syndemically interacted with different sources of vulnerabilities, enhancing the deleterious effects of all factors on the health of the population^25^.

Nonetheless, the implementation of the PNVS is an agenda that gathers the three levels of SUS management and social control, but the challenges are great and numerous. For instance, the compartmentalization of knowledge, processes, and practices hinders an effective integration of different surveillance practices^11^. Organizational barriers and the distance from the territory limit the effective articulation between the healthcare network and surveillance. Barriers to effective community participation in social control, insufficient funding, and absence of indicators for evaluation and monitoring of PNVS implementation, included in SUS management instruments, are additional elements that contribute to this panorama.

Most of these challenges depend on structural changes and organizational culture that take place outside the scope of direct attributions of education and research institutions that develop activities in the field of epidemiology. However, the PNVS highlights a number of needs that can and should be contemplated by epidemiology, among which the following stand out^10^:

- production of evidence from the analysis of the health situation to subsidize health management;
- investigation of outbreaks and unusual events or health situation arising from environmental impacts of productive processes and activities;
- identification of conditions and determinants of health in the territory;
- assessment of the impact of new health-related technologies and services in order to prevent risks and adverse events;
- adoption of epidemiological criteria for identifying and establishing priority lines of research; and
- development of health workforce qualification and training actions in the field of epidemiology and health surveillance.

These needs provide opportunities for greater interaction between epidemiology and health policies, programs, and services, as advocated by the Fifth Strategic Plan of Epidemiology^7^. The success of this endeavor depends, to some extent, on the updating of epidemiology training models, either in undergraduate or graduate studies; on a greater appreciation for the activities of knowledge translation^8^; and on a greater updating of the academic environment in terms of advances in health surveillance policies in Brazil.

### Health surveillance in research and graduate studies

Health surveillance is a fundamentally interdisciplinary activity, but has epidemiology as one of its main axes of knowledge and practice. Mainly until the 1980s, when graduate studies in Collective Health in Brazil deepened its institutionalization process, the emphasis of epidemiology was less on its scientific aspect and more on issues related to the field of epidemiology applied to health services, considering the process of the creation and consolidation of the SUS.

The expansion of the Brazilian National Graduate System had unquestionable effects on the training of professors and researchers and on the expansion and qualification of scientific production in several areas of knowledge^26^. In 2024, there were almost 100 graduate programs in Collective Health in operation in 22 Federative Units, almost all with areas of concentration and/or lines of research in Epidemiology. In the same year, the graduate programs accounted for almost 10 thousand students, including enrolled or graduated, in addition to more than two thousand professors. Overall, the process of expansion of graduate programs in Collective Health reflected in the expansion of the base of Epidemiology researchers and professors, contributing to the growth and improvement of Brazilian scientific production in this area, which may also have been reflected in the presence of research lines and projects, theses and dissertations, and articles in journals on the topic of health surveillance.

To evaluate the insertion of the topic of health surveillance in the scope of Brazilian scientific research, a search of scientific articles listed in the PubMed database was carried out, in which the term "surveillance" was mentioned in the title or in the descriptors in health science (DeCS/MeSH) and at least one of the authors was affiliated with a Brazilian institution, without date restriction. Preprint manuscripts and those without abstract were excluded. Thus, a total of 1,575 articles in which the topic of surveillance was mentioned, produced with the participation of at least one author affiliated with a Brazilian institution, were found. This set of articles is, here, freely called "surveillance articles with Brazilian authors." For the analysis of the data derived from this search, an eight-point cubic splines method for temporal trend curves smoothing was used. To present the terms (tags; n=21,756) mentioned in the articles, the word cloud technique was used. To this end, the terms used to better characterize the topics were harmonized and grouped. Terms related to age, sex, and species (human or animal) were excluded. This process was freely carried out by the author, according to his experience and vision on the subject, resulting in a listing of 16,309 terms (75% of the total). The perspective provided by this approach has as unit of analysis not the article, but the terms.

**Figure 1:**
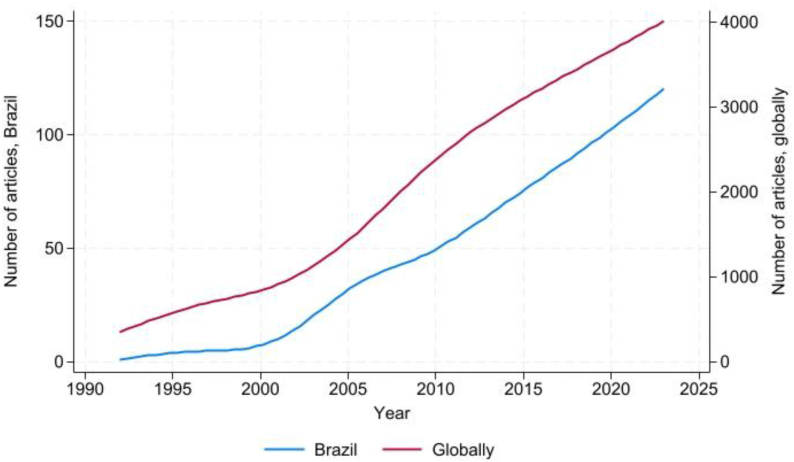
Temporal trend of scientific articles addressing the topic of surveillance available in the PubMed portal, in Brazil and globally, 1990–2024.

In Figure 1, we show the temporal trend of articles addressing the topic of surveillance, globally and with at least one of the authors affiliated with a Brazilian institution. We observe that trends are similar, with greater acceleration in the 2000s.

**Figure 2:**
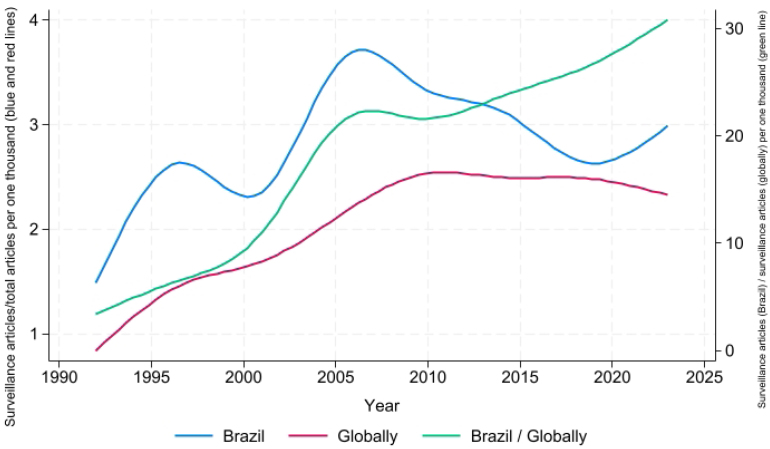
Temporal trend of the ratio between articles addressing the topic of surveillance and the total of articles registered in the PubMed portal, globally (red line) and with at least one of the authors affiliated with a Brazilian institution (blue line), and the ratio between surveillance articles in Brazil and globally (green line), 1990–2024.

In Figure 2, we show the temporal trend of the ratio between articles addressing the topic of surveillance and the total of articles registered in the PubMed portal, globally and with at least one of the authors affiliated with a Brazilian institution, in addition to the ratio between surveillance articles in Brazil and the global total. For Brazil, despite the variations, we observed that surveillance studies represent a growing share of the total of articles published until 2005, subsequently dropping until a new increase as of the beginning of the Covid-19 pandemic. Globally, there is an increase in the participation of total surveillance articles until 2010, when the curve stabilizes. Considering the curve of the ratio between surveillance articles published in Brazil and globally, the share of surveillance articles published by Brazilian authors in relation to the global total has been growing in a sustained way, despite representing a relatively low value (about 30 surveillance articles of national authors per one thousand surveillance articles published globally at the end of the series).

As per Figures 1 and 2, although the annual volume of surveillance articles with national authors and the ratio in relation to the global total are relatively low, the trend is of sustained increase for both indicators, suggesting a greater emphasis on scientific production in this field. This phenomenon may have been fostered by a greater interest in research to strengthen health surveillance actions due to the increasing risk of the emergence of new pathogens^27^.

**Figure 3:**
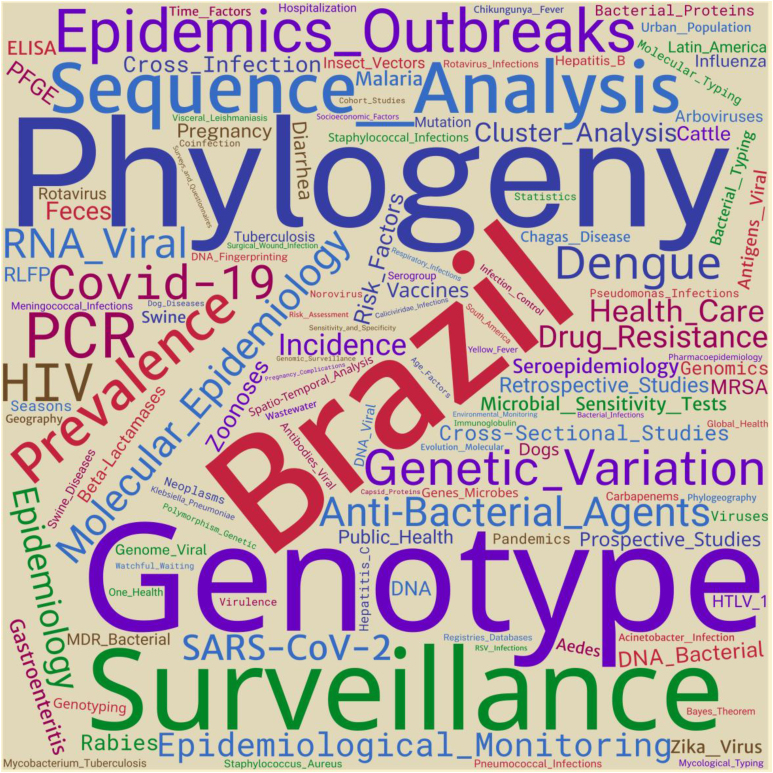
Cloud of terms (tags) mentioned in the selected articles, after harmonization process, which had at least 19 occurrences.

Identifying the role of epidemiology in the growing scientific production of Brazilian authors on surveillance is not an obvious task, due to the lack of objective markers available in the databases concerning which scientific contribution could be effectively classified as coming from the field of Epidemiology. This issue is deepened in Figure 3, although indirectly, by presenting the relative frequency of the terms (tags) mentioned in the articles on surveillance, after the harmonization process, which presented at least 19 occurrences.

The panorama presented in Figure 3 reinforces the interdisciplinary character in health surveillance, allowing us to identify comprehensive classes of topics. The class defined by the very use of the term "surveillance" or related practices and elements (e.g., vaccines, databases, and records) represented 6% of the mentions. Classes defined according to areas of knowledge, such as Epidemiology (e.g., disease frequency measures, study designs, and data analysis) and Biology (e.g., genotype, phylogeny, genetic variation, and entomology), obtained about 21% of the mentions each. Another high-frequency class refers to specific diseases and/or infectious agents (e.g., Covid-19, HIV, dengue, zoonoses, and SARS-CoV-2; 20%). Other identified classes were the issues related to treatment and health care (e.g., health care, antibacterial agents, and antimicrobial resistance; 8%), the techniques commonly used in diagnosis (e.g., PCR and ELISA; 3%), and geographical location (e.g., Brazil and Latin America; 11%).

These results should be interpreted with caution, given the relative informality of the classification employed. Nevertheless, it can be understood that, overall, authors of the analyzed articles address surveillance topics and use methods and techniques of interest to this area, but rarely focus on surveillance itself, its processes, and practices. Still, there is little representativeness of noncommunicable diseases in this context, possibly due to the emphasis on studies in contexts of outbreaks and pandemics. Epidemiology, Clinics, and Biology stand out as areas of knowledge that most directly contributed to surveillance studies.

The insertion of surveillance as an object of study in graduate programs in Collective Health in Brazil is not as expressive as one could imagine, considering that virtually all programs develop lines of research in Epidemiology. In 2023, of the 97 graduate programs with data available on the Sucupira Platform, two of them (one academic and one professional) had the term "surveillance" in their denomination. Of the 509 lines of research registered, 18 (3.5%) had the term "surveillance" in the title, while the mention was made in the title of 214 (3.2%) of the 6,680 research projects listed. The presence of the term "surveillance" in the title of research projects was more frequent in academic programs (3.6%) than in professional ones (2.7%). Regarding dissertations and theses, among the 2,026 listed, 43 (2.1%) had the term "surveillance" in the title, 57 (2.8%) in the keywords, and 180 (8.8%) in the abstract. The main topics, extracted from the title of dissertations and theses, were: vector-borne diseases (n=9), food and nutrition (n=6), respiratory infections (n=5), popular participation and health education (n=5), articulation between surveillance and healthcare network (n=5), and noncommunicable diseases and events and vital events (n=5). The main keywords listed were health surveillance/public health surveillance (n=19), epidemiological surveillance (n=8), food and nutritional surveillance (n=6), and occupational health surveillance (n=5).

Naturally, the absence of the term "surveillance" in titles, keywords, and abstracts does not necessarily mean that the topics or objects of dissertations and theses do not have implications or are irrelevant to health surveillance. Perhaps they are just studies that do not "recognize" themselves as related to surveillance, an unusual aspect, as the scope of health surveillance, as explained in the PNVS, is sufficiently broad to encompass multiple approaches. For instance, the surveillance of chronic noncommunicable diseases is based on methods other than those usually employed for acute communicable diseases. In this case, sequential population surveys and vital statistics analysis play a key role. Likewise, the surveillance of chronic communicable infections, such as tuberculosis and HIV/AIDS, uses approaches that require close connections to the healthcare network and the monitoring and evaluation of the care provided to people living with these conditions. The articulation between health surveillance, healthcare network, and health promotion and protection actions is explicitly provided for in the PNVS and is fundamental to achieve integrality in the care of chronic diseases. Considering the recommendations of the PNVS and the National Primary Care Policy (*Política Nacional de Atenção Básica* – PNAB), health surveillance is a structuring axis of Primary Health Care, implying that the integration between surveillance, care, and health promotion and protection actions is indispensable in the context of SUS^22^.

Even in the field of communicable diseases, more typically associated with surveillance, studies on vaccination and vaccination coverage do not always take the principles and practices of health surveillance as a reference for analysis. Although mental health assessment is an important issue for occupational health surveillance, the link between these studies and surveillance actions is not always well explained. The analysis of health situation and laboratory surveillance, although considered transversal areas of health surveillance, are not always thus identified. Even studies on the determinants of the health-disease process are essential for risk assessment, especially in vulnerable populations. Taking this into consideration, we may say that not everything is surveillance, but much of what is produced in epidemiology could be found in surveillance, being necessary to develop specific actions within the scope of graduate programs in Collective Health so that these interrelationships are strengthened.

### Challenges and opportunities

The publication of the Fifth Strategic Plan for the Development of Epidemiology in Brazil (2025–2029), after almost two decades since the publication of the IV Plan, takes place in a national and global context of great transformations, which impose new challenges to guarantee the heath of the Brazilian population^7^.

The deepening of transnational financial capitalism generates structural vectors of production of population illness, including increasing inequality and poverty, stimulating the consumption of products harmful to health — such as alcohol, tobacco, and ultra-processed foods —, precarious working conditions, reduction of social protection policies, the expansion of pollution, and the intensification of the climate crisis^28,29^. In this context, it is increasingly challenging to strengthen multilateral solidarity relations for the preparation and response to health emergencies and the climate crisis.

Difficulties in finding solutions become even greater with the arising of anti-science feeling in society, in particular of vaccine hesitancy, including among health professionals^30,31^. This may have an additional impact, as a positive action would be expected from these professionals to resolve doubts and contribute to increased confidence in vaccines^32^.

Although there are considerable challenges and the implementation of effective actions is increasingly pressing, it is necessary to recognize that several local and global resistance initiatives and movements have produced substantial advances in various dimensions of public health. For example, changes in the International Health Regulation and the approval of the new pandemics treaty, although falling short of global needs, can be considered achievements and provide opportunities to move forward in the structuring of best practices for preparing for and responding to health emergencies^33,34^. In 2024 and 2025, several countries received certification for the elimination of infectious diseases as a public health issue, such as trachoma (seven countries), malaria (five countries), lymphatic filariasis (two countries), leprosy (one country), and African trypanosomiasis (three countries). In Brazil, after a period of reduction of vaccination coverage, we observed a reversal of this scenario in relation to several vaccines of the children's vaccine schedule and the increase in the number of municipalities meeting the goals set^35^.

Despite the greatness of these achievements, their sustainability depends on the success of the struggles in defense of democracy, life, and health as a universal right. It is with this commitment that the Fifth Strategic Plan for the Development of Epidemiology in Brazil (2025–2029) systematizes a set of problems and proposes a series of actions to tackle them in the areas of training and research in epidemiology and the insertion of epidemiology in health policies, programs, and services.

Health surveillance is one of the main topics in the Fifth Strategic Plan for the Development of Epidemiology in Brazil (2025–2029). Some proposals are worth highlighting, such as: the creation of curriculum components of health surveillance and public health emergencies in undergraduate and graduate courses, fostering spaces for health surveillance practices, support for continuing education of health surveillance teams, fostering the implementation of research oriented toward the qualification of health surveillance actions, development of strategic indicators of territorial health surveillance, and the defense of the effective implementation of the PNVS^7^.

The Fifth Strategic Plan for the Development of Epidemiology in Brazil (2025–2029) is an essential vector for the theoretical, methodological, and practical development of epidemiology. In acknowledging health surveillance as a central topic for the development of epidemiology in the country, the Plan is also — and above all — a call for epidemiology committed to strengthening the SUS and developing successful and participatory strategies for reducing social inequalities in health.
