# Supplementary material for: Epidemiology and health surveillance: synergies and challenges in a changing world
Source: Rev Bras Epidemiol. 2026 Jan 26;28(Suppl 1):e250006supl1. doi: 10.1590/1980-549720250006.supl.1 (PMC12834469; doi:10.1590/1980-549720250006.supl.1)
Supplement: Translation

# Translation: Epidemiologia e vigilância em saúde: sinergias e desafios em um mundo em transformação

**DOI:** 10.1590/1980-549720250006.supl.1.2

**Authors:** Guilherme Loureiro Werneck

**Affiliations:** I Rio de Janeiro RJ Brasil Universidade do Estado do Rio de Janeiro, Instituto de Medicina Social – Rio de Janeiro (RJ), Brasil.; II Rio de Janeiro RJ Brasil Universidade Federal do Rio de Janeiro, Instituto de Estudos em Saúde Coletiva – Rio de Janeiro (RJ), Brasil.; III Brasília DF Brasil Ministério da Saúde, Secretaria de Vigilância em Saúde e Ambiente – Brasília (DF), Brasil.

**Keywords:** Vigilância em saúde pública, Epidemiologia, Aplicações da epidemiologia, Capacitação profissional, Pesquisas sobre serviços de saúde

## Abstract

Este ensaio discute os desafios concernentes à interação da epidemiologia com políticas, programas e serviços de saúde, com destaque para a vigilância em saúde. Ainda que o escopo dela compreenda saberes para além da epidemiologia, a utilização do conhecimento epidemiológico é elemento fundamental para a qualificação das suas ações. Apresenta-se a trajetória do processo de institucionalização da vigilância em saúde no Brasil, que culmina com a instituição da Política Nacional de Vigilância em Saúde (PNVS) em 2018. A PNVS destaca uma série de necessidades que podem e devem ser contempladas pela epidemiologia e que oferecem oportunidades para a maior interação entre epidemiologia e políticas, programas e serviços de saúde, mas exige uma atualização dos modelos de formação em epidemiologia, seja na graduação ou na pós-graduação, uma maior valorização das atividades de tradução do conhecimento e uma maior atualização do ambiente acadêmico em relação aos avanços nas políticas de vigilância em saúde do Brasil. Com base na análise de artigos brasileiros sobre vigilância em saúde listados no PubMed e em dados de linhas, projetos de pesquisa e trabalhos de conclusão de curso de programas de pós-graduação, avalia-se a inserção do tema da vigilância em saúde na pesquisa e pós-graduação em saúde coletiva no Brasil. Conclui-se discutindo a contribuição do V Plano Diretor para o Desenvolvimento de Epidemiologia no Brasil (2025-2029) para o fortalecimento da vigilância em saúde.

## INTRODUÇÃO

O arcabouço teórico da epidemiologia toma a distribuição populacional do processo saúde-doença e de seus determinantes e limitantes como domínio particular de interesse^1,2^. Nesse contexto, a epidemiologia trabalha com teorias sobre os mecanismos específicos causadores desses eventos e sobre os motivos que levam às heterogeneidades espaço-temporais de sua distribuição nas populações^2^, sutil diferença que se expressa em múltiplas práticas e perspectivas de epidemiologia, que, por sua vez, refletem visões de mundo vigentes em distintos tempos e lugares^3^.

Reconhecer tal diversidade implica demarcar a concepção de epidemiologia aqui empregada: a que parte do princípio de que a epidemiologia é um empreendimento científico que busca a geração de conhecimento inovador e se compromete com sua tradução e aplicação para subsidiar ações e políticas efetivas que levem à redução das iniquidades sociais em saúde e que promovam uma sociedade mais justa^2^. Uma epidemiologia que reconhece suas raízes nas lutas pela democratização do país e pela Reforma Sanitária, que não se conforma em ser somente epidemiologia-ciência, que necessita ser também epidemiologia-ação, enfim, uma epidemiologia da Saúde Coletiva^4^.

Encontra-se justamente nessa interface entre ciência e prática um dos maiores desafios para o desenvolvimento de uma epidemiologia implicada com a transformação social. Um primeiro entrave é a própria natureza e dinâmica das decisões em saúde pública. Mesmo que os diferentes níveis de gestão do Sistema Único de Saúde (SUS) possam reconhecer que tais decisões devem ser pautadas na melhor evidência científica disponível, é notório que elas ultrapassam o escopo da ciência, exigindo ponderações de cunho social, econômico, legal e ético, bem como de valores e princípios, como os de equidade, transparência e participação social^5^. Ainda assim, a epidemiologia tem sido cada vez mais frequentemente instada a contribuir nesse processo decisório, como se pôde observar durante a pandemia de Covid-19^6^. Essa recente experiência de ter sido alçada a um alto grau de protagonismo e prestígio revelou a maturidade técnica e científica da epidemiologia, assim como sua capilarização nas práticas das instituições de saúde. Entretanto, desvelou também uma série de desafios no que concerne à sua interação com políticas, programas e serviços de saúde^7,8^.

Considerando as identidades entre suas definições e objetivos, um campo de práticas inequívoco para a epidemiologia é a vigilância em saúde. A definição de epidemiologia abarca tanto o estudo do processo saúde-doença e dos seus determinantes em populações, como a aplicação desse conhecimento para controlar problemas de saúde^9^. Já a vigilância em saúde enfatiza o processo de transformação dos dados em informações para subsidiar o planejamento e a implementação de ações de proteção e promoção da saúde e de prevenção e controle de riscos, agravos e doenças em populações^10^. Ainda que o escopo da vigilância em saúde compreenda saberes para além da epidemiologia, o conhecimento epidemiológico é fundamental para a qualificação das suas ações^11^.

### Declaração da disponibilidade de dados

Todo o conjunto de dados que dá suporte aos resultados deste estudo está disponível mediante solicitação ao autor.

### Institucionalização da vigilância em saúde no Brasil

No início do século XX, mesmo no âmbito internacional, as práticas de vigilância não apresentavam significativa organização institucional. A expressão "vigilância epidemiológica" passou a ser adotada no controle das doenças transmissíveis por volta da metade do século XX, enfatizando inicialmente a vigilância de pessoas com base em medidas de isolamento ou de quarentena, mas logo ampliando seu escopo para eventos de saúde em populações. A estruturação e institucionalização de serviços especificamente orientados para a execução das ações de vigilância epidemiológica ganham força no cenário internacional com a criação da unidade de vigilância epidemiológica na Divisão de Doenças Transmissíveis da Organização Mundial da Saúde (1965) e com as discussões técnicas da 21ª Assembleia Mundial de Saúde (1968), que reforçam a concepção de vigilância como uma função essencial das práticas de saúde pública^12,13^. Em paralelo, o Regulamento Sanitário Internacional (RSI), criado em 1951, e suas subsequentes alterações, estabeleceram novos processos de monitoramento, vigilância e resposta às emergências de saúde pública de importância internacional^14^.

A constituição e institucionalização da vigilância em saúde no Brasil têm como um possível marco a criação do Departamento Nacional de Endemias Rurais (DNERu), em 1956, que ofereceu uma oportunidade de integração, em uma única instituição, das campanhas contra as chamadas grandes endemias, que abarcavam ações de epidemiologia e vigilância^15^. Porém, somente com a criação do Sistema Nacional de Vigilância Epidemiológica (SNVE), em 1975, é que se estabelece uma consolidação normativa sobre a organização das ações de vigilância epidemiológica no país. Fundamentadas em ações verticalizadas e centralizadas nas esferas de gestão federal e estadual da saúde, o SNVE se reorganizou a partir da década de 1990, com a criação e implementação do SUS e o estabelecimento do Centro Nacional de Epidemiologia (CENEPI).

Esse processo avançou nos anos subsequentes, a partir de marcos normativos como:

a Portaria 1.399/1999 (GM/MS), que estabelece as atribuições de cada esfera de governo na área de epidemiologia e controle de doenças e define a sistemática de financiamento^16^;

a criação, em 2003, da Secretaria de Vigilância em Saúde (SVS/MS) com o intuito de fortalecer e ampliar as ações de Vigilância em Saúde no Ministério da Saúde;

o Pacto pela Saúde (2006), em que o SNVE passou a ser denominado Sistema Nacional de Vigilância em Saúde (SNVS);

a Portaria 3.252/2009 (GM/MS), que estabeleceu as diretrizes para execução e financiamento das ações de Vigilância em Saúde no Brasil, ampliando seu escopo para abranger as vigilâncias epidemiológica, sanitária, em saúde do trabalhador e em saúde ambiental, além da promoção da saúde e vigilância da situação de saúde^17^.

O movimento pela estruturação de uma política do Estado brasileiro para a vigilância em saúde se fortalece com a publicação da Portaria 1.378/2013 (GM/MS), que prevê a criação do Grupo de Trabalho Tripartite para discussão e elaboração de uma Política Nacional de Vigilância em Saúde, o que é efetivado no mês seguinte^18,19^. Afinal, a PNVS foi instituída em 2018 por meio da Resolução 588/2018 do Conselho Nacional de Saúde, considerando as resoluções da 1ª Conferência Nacional de Vigilância em Saúde, realizada em Brasília/DF no início de 2018^10,20^.

### A Epidemiologia e a PNVS

A PNVS é uma política pública de Estado de caráter universal, transversal e orientadora do modelo de atenção à saúde nos territórios e que define os princípios, as diretrizes e as estratégias a serem observados pelas três esferas de gestão do SUS para o desenvolvimento da vigilância em saúde. A PNVS estabelece que a organização das ações da vigilância em saúde compreende a articulação dos saberes, dos processos e das práticas das vigilâncias epidemiológica, sanitária, em saúde ambiental, em saúde do trabalhador e trabalhadora. Ao mesmo tempo, a PNVS visa a integralidade na atenção à saúde e pressupõe a inserção da vigilância em saúde na rede de atenção à saúde. A PNVS contempla toda a população, mas prioriza territórios e populações em situação de maior risco e vulnerabilidade, de modo a superar desigualdades sociais e de saúde e buscar a equidade na atenção^10^.

A PNVS é fruto das lutas pela constituição e defesa do SUS, e o sucesso de sua implementação representa a consolidação de uma visão abrangente de vigilância em saúde como um alicerce fundamental da promoção e proteção à saúde da população e um eixo estruturante da atenção primária à saúde^21-23^.

Apesar de ter sido instituída em 2018, ainda não se observa uma efetiva implementação da PNVS nos territórios. Esses sete anos de vigência da PNVS foram particularmente desafiadores. O ciclo de austeridade fiscal associado à maior participação das emendas parlamentares no orçamento da saúde implicou agravamento do já crônico subfinanciamento do SUS e limitação na execução de políticas sociais^24^. Nesse contexto, o impacto da pandemia de Covid-19 foi dramático: aprofundou as já substanciais desigualdades sociais do país e interagiu sindemicamente com diferentes fontes de vulnerabilidades, potencializando os efeitos deletérios de todos os fatores na saúde da população^25^.

Que pesem essas intempéries, a implementação da PNVS é uma agenda que une os três níveis de gestão do SUS e o controle social, mas os desafios são grandes e de diversas matizes. Por exemplo, a compartimentalização de saberes, processos e práticas dificulta uma efetiva integração das diferentes vigilâncias^11^. Entraves organizacionais e o distanciamento do território limitam a efetiva articulação entre rede de atenção e vigilância. Barreiras a uma efetiva participação da comunidade no controle social, financiamento insuficiente e ausência de indicadores para avaliação e monitoramento da implementação da PNVS, inseridos nos instrumentos de gestão do SUS, são elementos adicionais que contribuem para esse panorama.

Boa parte desses desafios depende de mudanças estruturais e de cultura organizacional que se dão fora do âmbito de atribuições diretas das instituições de ensino e pesquisa que desenvolvem atividades no campo da epidemiologia. No entanto, a PNVS destaca uma série de necessidades que podem e devem ser contempladas pela epidemiologia, entre as quais destacam-se^10^:

- produção de evidências a partir da análise da situação da saúde para subsidiar a gestão em saúde;
- investigação de surtos e eventos inusitados ou situação de saúde decorrentes de impactos ambientais de processos e atividades produtivas;
- identificação dos condicionantes e determinantes de saúde no território;
- avaliação do impacto de novas tecnologias e serviços relacionados à saúde, de forma a prevenir riscos e eventos adversos;
- adoção de critérios epidemiológicos para a identificação e definição das linhas prioritárias de pesquisas; e
- desenvolvimento de ações de qualificação e capacitação da força de trabalho em saúde na área de epidemiologia e vigilância em saúde.

Essas necessidades oferecem oportunidades para a maior interação entre a epidemiologia e as políticas, e os programas e os serviços de saúde, nos moldes preconizados pelo V Plano Diretor para o Desenvolvimento da Epidemiologia no Brasil (2025–2029)^7^. O sucesso dessa empreitada passa, por um lado, pela atualização dos modelos de formação em epidemiologia, seja na graduação ou na pós-graduação, pela maior valorização das atividades de tradução do conhecimento^8^ e por uma maior atualização do ambiente acadêmico em relação aos avanços nas políticas de vigilância em saúde do Brasil.

### Vigilância em saúde na pesquisa e pós-graduação

A vigilância em saúde é uma atividade fundamentalmente interdisciplinar, mas que tem a epidemiologia como um de seus principais eixos de conhecimento e prática. Até, principalmente, a década de 1980, quando a pós-graduação em saúde coletiva no Brasil aprofundou seu processo de institucionalização, a ênfase da epidemiologia era menos no seu aspecto científico e mais nas questões relacionadas ao campo da epidemiologia aplicada aos serviços de saúde, tendo em vista o processo de criação e consolidação do SUS.

A expansão do Sistema Nacional de Pós-Graduação brasileiro teve efeitos inquestionáveis na formação de docentes e pesquisadores e na expansão e qualificação da produção científica em diversas áreas do conhecimento^26^. Em 2024, havia quase 100 programas de pós-graduação em Saúde Coletiva em funcionamento em 22 Unidades da Federação, quase todos com áreas de concentração e/ou linhas de pesquisa em Epidemiologia. Nesse ano, os programas de pós-graduação contavam com quase 10.000 alunos, entre matriculados ou titulados, além de mais de 2.000 docentes. De modo geral, o processo de expansão dos programas de pós-graduação em Saúde Coletiva refletiu na ampliação da base de pesquisadores e docentes em Epidemiologia, contribuindo para o crescimento e aprimoramento da produção científica brasileira nesse campo, o que deve ter se refletido também na presença de linhas e projetos de pesquisa, trabalhos de conclusão de curso e artigos em periódicos sobre o tema da vigilância em saúde.

Para avaliar a penetração do tema da vigilância em saúde no âmbito da pesquisa científica brasileira, procedeu-se a uma busca de artigos científicos listados no portal de busca PubMed, em que o termo "vigilância" aparecia no título ou nos descritores em ciência da saúde (DeCS/MeSH) e que pelo menos um dos autores registrou sua afiliação a uma instituição brasileira, sem restrição de data. Foram excluídos manuscritos em *preprints* e sem resumo. Assim, chegou-se a um total de 1.575 artigos nos quais o tema da vigilância foi mencionado, produzidos com a participação de pelo menos um autor afiliado à instituição brasileira. Esse conjunto de artigos é, aqui, livremente denominado "artigos de vigilância com autores brasileiros". Para a análise dos dados derivados dessa busca, usou-se o método de *cubic splines* com 8 bandas para suavizamento nas curvas de tendência temporal. Para apresentar os termos (etiquetas ou *tags*; n=21.756) registrados nos artigos, utilizou-se a técnica de nuvem de palavras. Para esse fim, procedeu-se à harmonização e agrupamento dos termos utilizados para melhor caracterizar temas. Foram eliminados termos referentes à faixa etária, sexo e espécie (humano ou animais). Esse processo foi realizado de forma livre pelo autor, de acordo com sua experiência e visão sobre o tema, resultando numa listagem de 16.309 termos (75% do total). A perspectiva propiciada por essa abordagem tem como unidade de análise não o artigo, mas os termos.

A Figura 1 mostra a tendência temporal dos artigos abordando o tema da vigilância, globalmente e com pelo menos um dos autores com afiliação a uma instituição brasileira. Observa-se que as tendências são similares, com maior aceleração na década de 2000.

**Figura 1:** Tendência temporal de artigos científicos abordando o tema da vigilância disponíveis no portal PubMed, Brasil e global, 1990–2024.

A Figura 2 mostra a tendência temporal da razão entre artigos abordando o tema da vigilância e o total de artigos registrados no portal PubMed, globalmente e com pelo menos um dos autores com afiliação a uma instituição brasileira, além da razão entre artigos de vigilância do Brasil e total global. Para o Brasil, apesar das variações, observa-se que os estudos de vigilância representam uma parcela crescente do total de artigos publicados até 2005, caindo em sequência até novo incremento a partir do início da pandemia de Covid-19. Globalmente, observa-se um incremento na participação de artigos de vigilância dentre o total até 2010, quando a curva se estabiliza. A curva da razão entre artigos de vigilância do Brasil e global mostra que a parcela de artigos de vigilância publicados por autores brasileiros em relação ao total mundial vem crescendo de forma sustentada, apesar de representar um valor relativamente baixo (cerca de 30 artigos de vigilância de autores nacionais por mil artigos de vigilância publicados globalmente no final da série).

**Figura 2:** Tendência temporal da razão entre artigos abordando o tema da vigilância e o total de artigos registrados no portal PubMed, globalmente (linha vermelha) e com pelo menos um dos autores com afiliação a uma instituição brasileira (linha azul), e da razão entre artigos de vigilância do Brasil e global (linha verde), 1990–2024.

As Figuras 1 e 2 indicam que, apesar de o volume anual de artigos de vigilância com autores nacionais e de a razão em relação ao total mundial serem relativamente baixos, a tendência é de aumento sustentado para ambos os indicadores, sugerindo uma maior ênfase na produção científica nesse campo. Esse fenômeno pode ter sido fomentado por um maior interesse em pesquisas para o fortalecimento das ações de vigilância em saúde em função do crescente risco de emergência de novos patógenos^27^.

Identificar o papel da epidemiologia na crescente produção científica de autores brasileiros sobre vigilância não é tarefa óbvia, devido à falta de marcadores objetivos e disponíveis nas bases de dados sobre que contribuição científica poderia ser efetivamente classificada como oriunda da área de epidemiologia. A Figura 3 explora essa questão, ainda que de forma indireta, mediante a apresentação da frequência relativa dos termos (etiquetas ou *tags*) registrados nos artigos sobre vigilância, após processo de harmonização, que apresentavam pelo menos 19 ocorrências.

**Figura 3:** Nuvem dos termos (etiquetas ou *tags*) registrados nos artigos selecionados, após processo de harmonização, que apresentavam pelo menos 19 ocorrências.

O panorama apresentado na Figura 3 reforça o caráter interdisciplinar na vigilância em saúde, permitindo identificar classes abrangentes de temas. A classe definida pelo próprio uso do termo "vigilância" ou de práticas e elementos a ela relacionados (p. ex., vacinas, banco de dados e registros) representou 6% das menções. Classes definidas de acordo com áreas de conhecimento, como a epidemiologia (p. ex., medidas de frequência de doenças, desenhos de estudo e análise de dados) e a biologia (p. ex., genótipo, filogenia, variação genética e entomologia), obtiveram cerca de 21% das citações cada uma. Outra classe de maior frequência refere-se às doenças e/ou agentes infecciosos específicos (p. ex., Covid-19, HIV, dengue, zoonoses e SARS-CoV-2; 20%). Outras classes identificadas foram as questões relacionadas ao tratamento e à atenção à saúde (p. ex., cuidados de saúde, agentes antibacterianos e resistência antimicrobiana; 8%), as técnicas comumente utilizadas em diagnóstico (p. ex., PCR e ELISA; 3%) e a localização geográfica (p. ex., Brasil e América Latina; 11%).

A interpretação desses resultados deve ser feita com cautela, dada a relativa informalidade da classificação empregada. Ainda assim, pode-se apreender que, em geral, os artigos analisados abordam temas e usam métodos e técnicas de interesse da vigilância, mas raramente estudam a própria vigilância, seus processos e práticas. Ainda, verifica-se a pouca representatividade de doenças e agravos não transmissíveis nesse contexto, possivelmente devido à ênfase nos estudos em contextos de surtos e pandemias. A epidemiologia, a clínica e a biologia se destacam como áreas de conhecimento que mais diretamente contribuíram para os estudos em vigilância.

A inserção da vigilância como objeto de estudo nos programas de pós-graduação em saúde coletiva no Brasil não é tão expressiva como se poderia imaginar, já que praticamente todos desenvolvem linhas de pesquisa em epidemiologia. Em 2023, dos 97 programas de pós-graduação com dados disponíveis na Plataforma Sucupira, 2 deles (1 acadêmico e 1 profissional) traziam o termo "vigilância" em sua denominação. Das 509 linhas de pesquisa cadastradas, 18 (3,5%) apresentavam o termo "vigilância" no título, enquanto a menção foi feita no título de 214 (3,2%) dos 6.680 projetos de pesquisa listados. A presença do termo "vigilância" no título dos projetos de pesquisa foi mais frequente nos programas acadêmicos (3,6%) do que nos profissionais (2,7%). Já em relação a teses e dissertações, entre as 2.026 listadas, 43 (2,1%) apresentavam o termo "vigilância" no título, 57 (2,8%) nas palavras-chave e 180 (8,8%) no resumo. Os temas principais, extraídos a partir do título das teses e dissertações, foram: doenças de transmissão vetorial (n=9), alimentação e nutrição (n=6), infecções respiratórias (n=5), participação popular e educação em saúde (n=5), articulação entre vigilância e rede de atenção à saúde (n=5) e doenças e eventos não transmissíveis e eventos vitais (n=5). As principais palavras-chave listadas foram vigilância em saúde/vigilância em saúde pública (n=19), vigilância epidemiológica (n=8), vigilância alimentar e nutricional (n=6) e vigilância em saúde do trabalhador (n=5).

Evidentemente, a ausência do termo "vigilância" em títulos, palavras-chave e resumos não significa, necessariamente, que os temas ou objetos dos trabalhos de conclusão de curso não tenham implicações ou não sejam relevantes para a vigilância em saúde. Talvez sejam apenas estudos que não "se reconheçam" como relacionados à vigilância, um aspecto inusitado, já que a abrangência da vigilância em saúde, nos termos explicitados na PNVS, é suficientemente ampla para acomodar múltiplas abordagens. Por exemplo, a vigilância de doenças crônicas e agravos não transmissíveis se apoia em métodos distintos daqueles usualmente empregados para as doenças transmissíveis de caráter agudo. Nesse caso, a realização de inquéritos populacionais sequenciais e a análise de estatísticas vitais desempenham papel fundamental. Da mesma forma, a vigilância das infecções crônicas transmissíveis, como tuberculose e HIV/aids, utiliza abordagens que requerem íntimas conexões com a rede de atenção e o monitoramento e a avaliação da cascata de cuidados das pessoas vivendo com essas condições. A articulação entre vigilância em saúde, rede de atenção e ações de promoção e proteção à saúde está explicitamente prevista na PNVS e é fundamental para alcançar a integralidade na atenção das doenças crônicas. Considerando o que preconiza a PNVS e a Política Nacional de Atenção Básica (PNAB), a vigilância em saúde é um eixo estruturante da atenção primária à saúde, implicando que a integração entre vigilância, atenção e ações de promoção e proteção à saúde é indispensável no contexto do SUS^22^.

Mesmo no campo das doenças transmissíveis, mais tipicamente associados à vigilância, os estudos sobre vacinação e cobertura vacinal nem sempre tomam os princípios e as práticas da vigilância em saúde como referencial de análise. Ainda que a avaliação da saúde mental seja tema caro à vigilância em saúde de trabalhadores e trabalhadoras, nem sempre o vínculo desses estudos com as ações de vigilância é bem explicitado. A análise de situação de saúde e vigilância laboratorial, embora sejam consideradas áreas transversais da vigilância em saúde, nem sempre são assim identificadas. Mesmo estudos sobre os condicionantes do processo saúde-doença são essenciais para a avaliação de risco, sobretudo em populações vulnerabilizadas. Tendo em vista todos esses aspectos, pode-se arriscar dizer que nem tudo é vigilância, mas muito do que se produz em epidemiologia poderia se encontrar na vigilância, restando que sejam desenvolvidas ações específicas no âmbito dos programas de pós-graduação em saúde coletiva para que essas interrelações sejam fortalecidas.

### Desafios e oportunidades

O lançamento do V Plano Diretor para o Desenvolvimento de Epidemiologia no Brasil (2025–2029), após quase duas décadas desde a publicação do IV Plano, se dá em um contexto nacional e mundial de grandes transformações, que impõem novos desafios à garantia da saúde da população brasileira^7^.

O aprofundamento do capitalismo financeiro transnacional gera vetores estruturais de produção de adoecimento populacional, incluindo o aumento da desigualdade e da pobreza, o estímulo ao consumo de produtos nocivos à saúde, como álcool, tabaco e ultraprocessados, a precarização das condições de trabalho, a redução das políticas de proteção social, a ampliação da poluição e a agudização da crise climática^28,29^. Nesse contexto, torna-se cada vez mais desafiador o fortalecimento das relações multilaterais solidárias para preparação e resposta a emergências sanitárias e à crise climática.

As dificuldades para encontrar soluções se tornam ainda maiores com a penetração do sentimento anticiência na sociedade, em particular da hesitação vacinal, inclusive entre profissionais de saúde^30,31^, o que pode ter um impacto adicional na medida em que esses profissionais seriam aqueles dos quais se esperaria uma ação positiva para dirimir dúvidas e contribuir para o aumento da confiança em vacinas^32^.

Ainda que os desafios sejam imensos e a implementação de ações efetivas seja cada vez mais premente, é preciso reconhecer que diversas iniciativas e movimentos de resistência locais e globais têm produzido avanços substanciais em várias dimensões da saúde pública. Por exemplo, as alterações no Regulamento Sanitário Internacional e a aprovação do novo tratado de pandemias, embora muito aquém das necessidades globais, podem ser consideradas conquistas e constituem oportunidades para avançar na estruturação de melhores práticas de preparação e resposta às emergências sanitárias^33,34^. Em 2024 e 2025, diversos países receberam a certificação de eliminação de doenças infecciosas como problema de saúde pública, como tracoma (7 países), malária (5 países), filariose linfática (2 países), hanseníase (1 país) e tripanossomíase africana (3 países). No Brasil, após um período de redução das coberturas vacinais, observou-se uma reversão desta para diversas vacinas do calendário infantil e o aumento do número de municípios, cumprindo as metas estabelecidas^35^.

Que pese a grandeza dessas conquistas, sua sustentabilidade depende do sucesso das lutas em defesa da democracia, da vida e do direito universal à saúde. É com esse compromisso que o V Plano Diretor para o Desenvolvimento da Epidemiologia no Brasil (2025–2029) sistematiza um conjunto de problemas e propõe uma série de ações para seu enfrentamento nas áreas de formação e pesquisa em epidemiologia e na inserção da epidemiologia em políticas, programas e serviços de saúde.

A vigilância em saúde é um dos temas privilegiados no V Plano Diretor para o Desenvolvimento da Epidemiologia no Brasil (2025–2029). Destacam-se proposições como a criação de componentes curriculares de vigilância em saúde e de emergências de saúde pública nos cursos de graduação e pós-graduação, estímulo a espaços de práticas em vigilância em saúde, apoio à educação permanente das equipes de vigilância em saúde, estímulo à implementação de pesquisas orientadas para a qualificação das ações da vigilância em saúde, desenvolvimento de indicadores estratégicos da vigilância em saúde de base territorial e defesa da implementação efetiva da PNVS^7^.

O V Plano Diretor para o Desenvolvimento da Epidemiologia no Brasil (2025–2029) é um vetor essencial para o desenvolvimento teórico, metodológico e prático da epidemiologia. Ao reconhecer a vigilância em saúde como um tema central para o desenvolvimento da epidemiologia no país, o Plano é, também e sobretudo, um chamado para uma epidemiologia comprometida com o fortalecimento do SUS e com a construção de estratégias exitosas e participativas para a redução das desigualdades sociais em saúde.
